# 

**Published:** 1847-06

**Authors:** 


### INDEX.

PAGE.

A

Abscess of the Jaws and its Treatment, by Dr. S. P. Hullihen, 138
Abscess of Antrum Maxillare, . 192
Accidents in the Extraction of Teeth, . . . . . . 109
Address, Opening, . . . 112
Address to the Members of the Am. Soc. of Dental Surgeons, by E. Parmly, M. D., D. D. S., 181
Address, Valedictory, before the Graduating Class of Baltimore College of Dental Surgery, by A. Westcott, M. D., . . . 202
Address to the Graduating Class of Balt. Col. of Dent. Surgery, by E. Parmly, M. D., D. D. S., 297
Alcoholic Fluids, Digestion of, 391
American Society of Dental Surgeons, Seventh Annual Meeting of, . . . . . . . 89
American Society of Dental Surgeons, Eighth An. Meeting of, 396
Annual Announcement of Jefferson Medical College of Phila., 115
Annual Announcement of the Med. Dep. of the Pa. College, 120
Arkansas Oil-Stone, . . . 110, 293
Artificial Teeth, Spiral Springs for, . . . . . . . 197
Atrophy following an Injury of the Right Superior Maxillary Nerve, . . . . . . 391

B

Baltimore College of Dental Surgery, . . . . . 112, 282
Beautiful Case of Instruments, . 196
Bleeding from Leech Bites, Means of Arresting, . . . 329
Bradford, Gunning S., M. D., Introductory Lecture on Obstetrics and Diseases of Women and Children, . . . . 296
Bulley, F. A., Esq., Case of Extraction of Artificial Teeth from the Gullet, by, . . . . 134

PAGE.

C

Camphor on the Teeth, Injurious Effects of, . . . . . 391
Cancer of the Bones, . . 395
Caries of Superior Maxillare and Death, . . . . . 185
Caries of Left Temporal Bone, 389
Catalogue, Annual, of the Officers and Students of the Ohio College of Dental Surgery, . 118
Circular, Annual, of the Massachusetts Medical College, . 119
Club Foot and Diseases of the Antrum, by Dr. Michel, . 281
Coley, James Milman, M. D., Practical Treatise on the Diseases of Children, by, . . 117
Contributions to Operative and Mechanical Dentistry, by W. H. Elliot, D. D. S., . . 68
Contraction of the Lower Jaw, 87
Connecticut Society, Proceedings of the Annual Convention of the, . . . . . . 117
Congenital Fissure of the Palate, &c., by Charles W. Stearns, Esq., . . . . . 238, 377
Correction, . . . . 289
Cox, John Redman, M. D., Epitome of the Writings of Hippocrates and Galen, by, . 115

D

Darrach, W., M. D., Introductory Lecture on the Theory and Practice of Medicine, in the Pennsylvania College, by, . 295
Dental Physiology and Surgery, Lectures on, by John Tomes, Esq., . . . . . 3, 121
Dental Surgeons, Mississippi Valley Association of, 108, 195
Dental Surgeons, Virginia Society of, . . . 109, 199
Dental Surgery, Baltimore College of, . . . 112, 282
Dental Exostosis, . . 111

PAGE.

Dental Register, by H. Crane, D. D. S., . . . . . 119

Dental Literature, . . . 193

Dental Ring and Drill Socket, 198

Dentistry, Contributions to Operative and Mechanical, by W. H. Elliot, . . . . . 68

Dentistry, Works on Operative and Mechanical, . . . 290

Diagnosis of Neuralgia and Neuritis, . . . . . . . 280

Dictionary of Medical Science, by Robley Dungleson, M. D., 116

Digestion of Alcoholic Fluids, 391

Diseases of Children, Practical Treatise on, by James Milman Coley, M. D., . . . . 117

Diseases and Operations of the Teeth, Lectures on, by Edwin Saunders, Esq., . . . 260

Drill-Stock, A New, . . . 108

Dungleson, Robley, M. D., Introductory Lecture by, . 118

Dwinelle, W. H., Dissertation on the Preparation of a Cavity in a Tooth, preparatory to Plugging, by, . . . . 74

Dwinelle, Dr., Letter from, . 375

E

Elliot, W. H., D. D. S., Contributions to Operative and Mechanical Dentistry, by, . . 68
Enlargement of the Journal and Library of Dental Science, . 106
Ethereal Vapor, Inhalation of, for inducing Insensibility to Pain of Surgical Operations, 194
Ethereal Vapor, for producing Insensibility to Pain of Surgical Operations, by A. Hill, D. D. S., . . . . . 224
Ethereal Vapor, Insensibility produced by, . . . . 230
Ethereal Vapor, . . . . 396
Exostosis, Dental, . . . 111
Extraction of Teeth, Accidents in the, . . . . . . 109
Extraction of Artificial Teeth from the Gullet, by F. A. Bulley, Esq., . . . . . 134

F

Fibrous Tumor of Upper Jaw, 77

PAGE.

File Carriers, . . . 291
Filling Teeth, . . . . . 110
Fœtus, with Left Arm partly amputated by the Funis coiled round it, . . . . . 280
Forceps for Extracting Upper Molar Roots, when not separated, . . . . . . 288

H

Hæmorrhage, Fatal, from a Free Incision of the Gums, . . 182
Hill on Inhalation of Ethereal Vapor, . . . . . . 224
Hippocrates and Galen, the Writings of, epitomised by John Redman Cox, M. D., . . 115
Hoston, Robert M., M. D., Introductory Lecture on Materia Medica, by, . . . . 293
Hullihen, Dr. S. P., on Abscess of the Jaws, and its Treatment, 138
Hullihen's, Dr. S. P., Uvula Scissors, . . . . . 274

I

Ibrahim Pacha and his Doctor's Bill, . . . . . . . 88
Imperfection of Speech, consequent upon Congenital Fissure of Soft Palate, New Instrument to remedy, by C. H. Stearns, Esq., . . . . . . . 234
Importance of Medical and Surgical Knowledge to the Dentist, by Jas. Robinson, D.D.S., 324
Incisor Teeth, Exchange of the Deciduous for the Permanent, by Wm. Lintott, Esq., . . 271
Inflamed Eyes, Application of Poultices to, . . . . 278
Inhalation of Ethereal Vapor for Inducing Insensibility to Pain of Surgical Operations, . . 194
Injurious Effects of Camphor on the Teeth, . . . . . 392
Instruments, Beautiful Case of, 196
Insane, Pa. Hospital for the, . 199
Insensibitity produced by Ethereal Vapor, . . . . . 230
Introductory Lecture, by Robley Dungleson, M. D., . . 118
Introductory Lecture, by A. Westcott, M. D., D. D. S., . 152

PAGE.
Introductory Lecture on Materia Medica, &c., in Jefferson Medical College, by Robert M. Hoston, M. D., . . . . 293
Introductory Lecture on the Theory and Practice of Medicine, in Pa. College, by W. Darrach, M. D., . . . . 295
Introductory Lecture on Obstetrics, and Diseases of Women and Children, in the Medical Dep. of University of N. Y., by Gunning S. Bradford, M. D., 296
Irregularity of the Teeth, . . 197

J

Jaw, Fibrous Tumor of the Upper, . . . . . . . 77

Jaw, Contraction of the Lower, 87

Jefferson Medical College, Annual Announcement of, . 115

Journal and Library of Dental Science, Enlargement of, . 106

L

Lectures on Dental Physiology and Surgery, by John Tomes, Esq., . . . . . 3, 121
Leech Bites, Means of Arresting Bleeding from, . . . . 329
Letter from Dr. W. H. Dwinelle, 375
Lintott, Wm., Esq., on the Exchange of the Deciduous for the Permanent Incisor Teeth, 271

M

Massachusetts Medical College, Annual Circular of, . . 119
Maxillare, Superior, Caries of, and Death, . . . . . 185
Meeting, Eighth Annual, of the Am. Soc. of Dental Surgeons, 396
Meeting, Annual, of the Pa. Ass'n of Dental Surgeons, . 276
Mississippi Valley Association of Dental Surgeons, . 108, 195

N

National Medical Convention, Minutes of the Proceedings of, 117
Neuralgia treated by Quinine and its Preparations, . . 277

PAGE.
Neuralgia and Neuritis, Diagnosis of, . . . . . . 280
Neuralgia, on the Treatment of, by Tobacco, . . . . 323
New Drill-Stock, . . . 108
New Method of Treating a Professional Rival, . . . 389

O

Obituary, . . . . 120, 200
Offer of a Bribe for a Good Review by a Dentist, . . . 390
Ohio College of Dental Surgery, Annual Catalogue of Officers and Students of, . . . 118
Oil-Stone, Arkansas, . 110, 293
Opening Address, . . . 112

P

Parmly, Eleazar, M. D., D. D. S., Address to the Am. Society of Dental Surgeons, by, . . 181
Parmly, E., M. D., D. D. S., Address to the Graduating Class of the Balt. College of Dental Surgery, by, . . . . 297
Pennsylvania College, Annual Announcement of the Medical Department of, . . . 120
Pennsylvania Hospital for the Insane, . . . . . . 199
Pennsylvania Ass'n of Dental Surgeons, . . . . 109
Pennsylvania Ass'n of Dental Surgeons, Annual Meeting of, 276
Phosphate of Lime, on the Manner in which it enters into Organized Beings, . . . 279
Plugging, Preparation of a Cavity, Preparatory to, by W. H. Dwinelle, . . . . 74
Plugging Teeth, . . . . 193
Polypus of the Nose and Ear, . 388
Proceedings of Last Meeting, Review of, . . . . 102
Proceedings of the National Medical Convention, . . 116
Proceedings of the Annual Convention of the Connecticut Society, . . . . 117
Professional Rival, New Method of Treating a, . . . 389

PAGE.

R

Review of Proceedings of Last Meeting, . . . . . 102
Robinson, James, D. D. S., on the Surgical, Mechanical and Medical Treatment of the Teeth, . . . . . . 113
Robinson, James, D. D. S., on the Importance of Medical and Surgical Knowledge to the Dentist, . . . . . 324

S

Saunders, Edwin, Esq., on the Diseases and Operations of the Teeth, . . . . . . 260
Saunders on the Teeth a Test of Age, . . . . . . 330
Spiral Springs, for Artificial Teeth, . . . . . . 197
Stearns', C. H., Esq., New Instrument for Imperfection of Speech, . . . . . 234
Stearns on Congenital Fissure of the Palate, Articulation, and Impediments of Speech, 238, 377
Subscribers, to, . . . . 112
Suffocation in an Infant, from Retraction of the Base of the Tongue, connected with Defect of Frænum, . . . . 189
Sulphocyanogen in Human Saliva, the Presence of, . . . 84
Surgeon Dentists, Pa. Ass'n of, 109
Surgical, Mechanical and Medical Treatment of the Teeth, by by James Robinson, D. D. S., 113
Swallowing a "Precious Morsel"—a Plate and Eleven Teeth, . . . . . . 294

T

Teeth, Filling, . . . . . 110
Teeth, Irregularity of, . . . 197
Teeth a Test of Age, by Edwin Saunders, D. D. S., . . . 330
Tobacco, on the Treatment of Neuralgia by, . . . . 323
Tomes, John, Esq., on Dental Physiology and Surgery, 3, 121
To Subscribers, . . . . 112
Transposition of Teeth, . . . 111
Trismus, Case of, . . . . 393

U

Uvula Scissors, a New Instrument, by Dr. S. P. Hullihen, 274

V

Virginia Society of Dental Surgeons, . . . . . 109, 199
Visit to Washington, . . . 284

W

Westcott, A., M. D., D. D. S., Introductory Lecture by, . 152
Westcott, A., M. D., D. D. S., Valedictory Address by, . 202
Works on Operative and Mechanical Dentistry, . . . 290

### EDITORS' PROSPECTUS

OF THE

### American Journal and Library of Dental Science.

this Journal has become the organ of the American Society of Dental Surgeons, and as its has been somewhat modified, it may be considered as having entered upon a new period of xistence; and its former publishers congratulate themselves and the profession on its good e. Its perpetuity is now considered as settled. The profession will always need a periodical ed to its interests, and who is better able to conduct it, than the only regularly organized and sive association of dental surgeons in the world. We repeat, that we congratulate ourselves ur professional brethren on an event so auspicious to the science of dental surgery, as the ation of a national society and the transfer of the journal to that body. The work can now be arly issued, whether its circulation be co-extensive with the profession or not. If the subscrip- should be limited, the issues can be made to correspond with the demand, so that the expense e society may be kept wholly within its means.

e American Journal and Library of Dental Science, is now proposed to take an independent d, and will hereafter solicit no favours from those members of the profession who are opposed e dissemination of periodical information. To others who are favourably inclined to a commu- of knowledge, this journal will not fail to commend itself, as one of the most efficacious means posing the impositions of quackery, and imparting to honorable pretensions that kind of infor- on which their usefulness and talents should secure.

e journal will be regularly issued on the first of December, March, June and September, ally, at the enhanced price of five dollars per volume, payable in all cases in advance. The of subscription may be paid to the treasurer of the society, Dr. C. A. Harris, Baltimore or to either of the editors, or any of the publishers, collaborators or acknowledged agents of ournal. Advance payment is required by a special resolution, passed at the last meeting of the ty, and is necessary to protect it from pecuniary loss, a neglect of which has involved the shers of the first volume in unexpected embarrassment, if not in ultimate loss.

save postage, the agents in all the large cities of the United States, and Great Britain, will be shed with copies to supply subscribers, by such conveyance as they shall prescribe. And e it can be done, even individuals may receive their numbers by private conveyance if they se.

th these few explanatory remarks, we commit our work to its coming destiny, after assuring rofession that no efforts will be spared to render it every way worthy of the great subject to h its pages will be exclusively devoted.

CHAPIN A. HARRIS,
AMOS WESTCOTT,
EDWIN J. DUNNING.

---

Besides the Collaborators, the following gentlemen are regularly authorized

### AGENTS OF THIS JOURNAL.

- [illegible]N & KELTS,..........Boston.

- M'CONNELL, M. D.....Richmond, Va.

- LAIRD, M. D.........Columbus, Ga.

- PARMLY, M. D.......New Orleans.

- cDONALD, M. D ...... Macon, Ga.

- GODDARD and FERGUSON Louisville, Ky.

- TAYLOR, M. D.. ....Cincinnati, Ohio.

- NCAMP, D. D. S,......Nashville, Tenn.

- DWIN, D. D. S.. ......Alabama.

- McCurdy, ............Philadelphia.

- NICHOLAS CLUTE,..... ....Louisville,

- BENJ. STRICKLAND,.........Cleveland, Ohio.

- B. B. BROWN, M. D.........St. Louis, Mo.

- W. R. SCOTT, D. D. S.........Raleigh, N. C.

- S. M. SHEPHERD,.............Petersburg, Va.

- JOHN A. CLEVELAND,........Charleston, S C.

- WM. F. BASON, D. D. S.,......N. Carolina.

- JOHN LEES, 23 Exchange Arcade, Manchester, England.

- Dr. E. H. Pebles, ..........Lexington, Mo.

